# MERGE: A Multinational, Multicenter Observational Registry for Myeloproliferative Neoplasms in Asia, including Middle East, Turkey, and Algeria

**DOI:** 10.1002/cam4.3004

**Published:** 2020-04-30

**Authors:** Mohamed A. Yassin, Ali Taher, Vikram Mathews, Hsin‐An Hou, Tahir Shamsi, Tülin Firatli Tuğlular, Zhijian Xiao, Soo‐Jeong Kim, Wu Depei, Junmin Li, Gerd Rippin, Islam Sadek, Asif Siddiqui, Raymond S. Wong

**Affiliations:** ^1^ National Center for Cancer Care and Research Hamad Medical Corporation Doha Qatar; ^2^ Department of Internal Medicine American University of Beirut Medical Center Beirut Lebanon; ^3^ Department of Haematology Christian Medical College Vellore India; ^4^ Division of Hematology Department of Internal Medicine National Taiwan University Hospital Taipei Taiwan; ^5^ Research Department National Institute of Blood Disease and Bone Marrow Transplantation Karachi Pakistan; ^6^ Department of Hematology Marmara University Hospital İstanbul Turkey; ^7^ MDS and MPN Centre Institute of Hematology & Blood Diseases Hospital CAMS and PUMC Tianjin China; ^8^ Severance Hospital Seoul Republic of Korea; ^9^ Department of Hematology The First Hospital Affiliated to Soochow University Suzhou China; ^10^ Ruijin Hospital Shanghai China; ^11^ IQVIA™ Neu‐Isenburg Germany; ^12^ Novartis Pharmaceuticals Corporation East Hanover NJ USA; ^13^ Novartis AG Basel Switzerland; ^14^ Sir YK Pao Centre for Cancer & Department of Medicine and Therapeutics The Chinese University of Hong Kong Prince of Wales Hospital Hong Kong SAR

**Keywords:** epidemiology, myeloproliferative disorders, neoplasms, quality of life

## Abstract

Philadelphia chromosome‐negative (Ph−) myeloproliferative neoplasms (MPNs) are a heterogeneous group of clonal disorders of the bone marrow, and are associated with a high disease burden, reduced quality of life (QOL), and shortened survival. This multinational, multicenter, non‐interventional registry “MERGE” was initiated with an objective to collect data on the epidemiological indices of classical Ph‐MPNs, existing treatment patterns, and impact of MPNs on health‐related QOL in various countries/regions in Asia, including the Middle East, Turkey, and Algeria. Of the 884 eligible patients with MPNs, 169 had myelofibrosis (MF), 301 had polycythemia vera (PV), 373 had essential thrombocythemia (ET), and 41 had unclassified MPNs. The median age was 58 years (range, 47‐66 years), and 50% of patients were males. The prevalence and incidence of MPNs were estimated to be 57‐81 and 12‐15 per 100 000 hospital patients per year over the last 4 years, respectively, in these countries. Total symptom score (mean [standard deviation; SD]) at baseline was highest in patients with MF (23.5 [17.47]) compared with patients with ET (14.6 [14.26]) and PV (16.6 [14.84]). Patients with ET had a lower mean (SD) number of inpatient visits (0.9 [0.77] days), and patients with MF had more outpatient visits (5.2 [3.17] days) on an average, compared with the entire MPN group. The study showed that patients with MPNs have a severe disease burden and reduced QOL. A discordance between physician and patient perception of symptom assessment was observed in this study (International clinical trials registry ID: CTRI/2014/05/004598).

## Graphical abstract

This MERGE registry study provides epidemiological data on the distribution of MPNs and their diagnosis, management, and treatment patterns in developing emerging countries in Asia, including the Middle East, Turkey, and Algeria. The study showed that patients with MPNs have a severe disease burden and reduced quality of life. A discordance between physician and patient perception of symptom assessment was observed in this study.

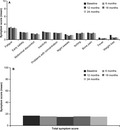

## 1 INTRODUCTION

The Philadelphia chromosome‐negative (Ph‐) classical myeloproliferative neoplasms (MPNs; including essential thrombocythemia [ET], polycythemia vera [PV], and myelofibrosis [MF]),^1^, ^2^ are generally associated with a substantial disease burden, often leading to a reduced quality of life (QOL) and shortened survival in many patients. Patients with MF generally have the highest symptom burden and shortest survival.^3^, ^4^ Most patients with MPNs suffer from pronounced symptom burden, which includes fatigue, pruritus, night sweats, microvascular symptoms, splenomegaly, and splenomegaly‐associated symptoms (eg, abdominal pain and early satiety).^4^, ^5^ Constitutional symptoms such as fever, night sweats, and weight loss are more frequently reported in patients with MF, whereas pruritus is more common in patients with PV.^6^ Recent data showed strong association between the MPN scoring system and disease phenotypes, progression, and survival.^7^ An MPN Symptom Assessment Form Total Symptom Score (MPN‐SAF TSS) ≥20, a worst individual item score > 5, or combined criteria of both warrant treatment.^8^

There has been a lack of estimates for the incidence/prevalence and treatment patterns of MPNs in many regions of the world, including countries from South Asia, Asia Pacific, Middle East, and Turkey, leading to a difficulty in estimating the true disease burden. Hence, there is a need to establish databases like registries, which would provide information on the “real‐world” data in these regions. This multinational, multicenter, noninterventional registry “MERGE” was initiated with an objective to collect data on the epidemiological indices of classical Ph‐MPNs, existing treatment patterns, and impact of MPNs on health‐related QOL (HRQOL) in various countries/regions in Asia, including the Middle East, Turkey, and Algeria. This primary descriptive analysis from the MERGE registry was performed to estimate the incidence/prevalence, natural disease course, and treatment patterns of MPNs in these countries/regions. There is limited real‐world evidence from many of the Asian countries, including the Middle East, regarding the impact of MPNs on HRQOL or the economic burden associated with these diseases. The secondary objective was to assess the impact of MPNs on HRQOL of patients in the MERGE registry and explore the association of HRQOL with the clinical outcomes and resource utilization.

## 2 METHODS

### 2.1 Study design

The MERGE registry (International clinical trials registry ID: CTRI/2014/05/004598) is a prospective observational study that included patients with MPNs diagnosed as per the World Health Organization (WHO) 2008 criteria. Patients who participated or were participating in a randomized clinical trial were authorized (Appendix 1). With the last protocol version, patients were planned to be followed up for 2 years (five visits over the course of study).

### 2.2 Key inclusion criteria

Male or female patients aged ≥ 18 years who had been diagnosed with MPNs according to the revised WHO criteria; patients willing and able to provide written informed consent according to local guidelines prior to any study procedures; patients whose historical medical records were available to verify the diagnosis; and patients literate in the language of the available HRQOL instrument and willing to complete the same, either alone or with minimal assistance from caregivers and/or trained site personnel were included in the study.

### 2.3 Key exclusion criteria

Any patient who was unavailable for long‐term follow‐up or with severe concomitant illnesses, according to the investigator's opinion, was excluded. Patients with an active alcohol or drug addiction that would have interfered with the patient's ability to comply with the study requirements were also excluded.

### 2.4 Assessments

The proportion of cases diagnosed with PV, ET, or MF over a year, among patients visiting MERGE sites in that time period was used as a proxy estimation of the prevalence of MPNs per 100 000 patients, by country. Similarly, the proportion of new cases diagnosed with PV, ET, or MF over a year, among patients visiting MERGE hospitals during the same period was used as a proxy estimation of the incidence of MPNs per 100 000 patients, by country. Both estimates were accompanied by 95% confidence intervals (CIs), using the Clopper and Pearson exact method.^9^ Disease transformation was summarized as the number and percentage of patients whose disease status changed from PV to post‐polycythemia vera MF (PPV‐MF)/acute myeloid leukemia (AML), ET to post‐ET MF/AML, and from MF to AML. Disease progression was defined according to clinician judgment: worsening of symptoms as per MPN‐SAF TSS, worsening of splenomegaly or hepatomegaly, and incidental finding on blood screen and/or bone marrow analysis. Analysis of treatment patterns was stratified by initial MPN diagnosis, type of progression, and line of therapy (LOT). The MPN‐SAF TSS was administered during the routine biannual follow‐up visits to assess the HRQOL. Information about MPN therapies was collected according to the investigator's decision within routine clinical practice. The MPN‐SAF TSS is a validated, concise, accurate tool to assess MPN symptom burden in patients with this disease.^3^, ^5^ It is a 10‐score questionnaire focusing on fatigue, concentration, early satiety, inactivity, night sweats, itching, bone pain, abdominal comfort, unintentional weight loss, and fever. In MERGE, MPN‐SAF TSS score was calculated only for patients who completed at least six of the 10 items on the MPN‐SAF TSS questionnaire. Both the physicians and patients assessed and reported MPN symptoms during the follow‐up visits. The level of inter‐rater agreement between physicians and patients on the proportion of symptomatic patients was assessed using Cohen's Kappa coefficient. The inter‐rated agreement was classified according to the categories defined by Landis et al^10^ Further, mixed effect model was applied to estimate the influence of the baseline variables (age, sex, weight, height, family history of MPNs, comorbidities, type of MPN initial diagnosis, baseline hemoglobin and platelet count, Eastern Cooperative Oncology Group performance status [ECOG PS], and time since diagnosis to treatment initiation) on the TSS score. Variables with a univariate *P*‐value < .20 were included in the multivariate model. Furthermore, medical resource utilization was assessed by evaluating inpatient and outpatient visits, hospice care, emergency room visits, and day care. Other parameters, such as spleen and blood parameters, were also assessed.

## 3 RESULTS

### 3.1 Patients

A total of 1044 patients with MPNs recruited from 54 sites in 17 countries were enrolled in the study (Appendix 1). Of these, 160 patients were excluded because of protocol deviations, primarily due to missing confirmed Ph‐MPN diagnosis as per WHO 2008 criteria.^11^ Although the diagnosis of MPNs was not confirmed as per WHO criteria, these patients were receiving therapies for MPNs. Overall, 884 eligible patients with MPNs were included, of which, 169 had MF, 301 had PV, 373 had ET, and 41 had unclassified MPNs (Table 1). Of the 884 patients, 422 (47.7%) discontinued before study completion, with the most common reasons for discontinuation reported as lost to follow‐up, withdrawal of consent, and other (Table 2). The median age was 58 years and 50% of the patients were male. Overall, 64% of patients had an ECOG PS of 0, 26% had an ECOG PS of 1, and 10% had an ECOG PS > 1. A total of 325 patients (36.8%) were diagnosed with or had experienced at least one comorbidity. The most frequently reported comorbidities were cardiovascular comorbidities (20.9%; including peripheral vascular disease [11.0%]) and diabetes without end‐organ damage (10.4%). The proportion of MF patients with cardiovascular comorbidities was considerably lower than that of ET and PV patients: 8.9% vs 20.9% and 20.1%, respectively. MF patients were less likely to have any type of insurance (64.5%) compared with ET patients (76.1%). Overall, 4.4% of patients with MPNs reported a history of non‐hematological malignancy, and 1.5% of patients reported a history of hematological malignancy.

**Table 1:** Patient baseline characteristics

Characteristics	ET (N = 373)	PV (N = 301)	MF (N = 169)
Age, median (Q1‐Q3), years	55.0 (41.0‐66.0)	60.0 (51.0‐67.0)	58.0 (50.0‐65.0)
≥65 y, n (%)	104 (27.9)	113 (37.5)	44 (26.0)
Male, n (%)	161 (43.2)	175 (58.1)	84 (49.7)
Time since diagnosis, median (Q1‐Q3), years	2.0 (0.5‐4.9)	2.4 (0.6‐5.2)	1.1 (0.3‐3.7)
Prognostic scoring classification at initial diagnosis, n (%)			
Low risk	143 (38.3)	121 (40.2)	23 (13.6)
Intermediate‐1 risk^a^	—	—	48 (28.4)
Intermediate‐2 risk^a^	—	—	45 (26.6)
High risk	92 (24.7)	119 (39.5)	24 (14.2)
Unclassified	134 (35.9)	55 (18.3)	29 (17.2)
Mutational status^b^, n (%)			
JAK2V617F	229 (61.4)	256 (85.0)	114 (67.5)
CALR^c^	9 (2.4)	0 (0.0)	0 (0.0)
JAK2 exon 12^c^	3 (0.8)	4 (1.3)	1 (0.6)
Unfavorable karyotype	80 (21)	57 (19)	59 (35)
Patients with comorbidities, n (%)	125 (33.5)	131 (43.5)	51 (30.2)
Comorbidities (in ≥ 10% of patients), n (%)			
Cardiovascular	75 (20.1)	79 (26.2)	15 (8.9)
Diabetes without end‐organ damage	32 (8.6)	33 (11.0)	19 (11.2)
At least one hematological malignancy	4 (1.1)	2 (0.7)	6 (3.6)
At least one non‐hematological malignancy	21 (5.6)	11 (3.7)	4 (2.4)
Nonmelanoma skin cancer	0 (0.0)	1 (9.1)	0 (0.0)
Prior history of thrombotic events	53 (14.2)	49 (16.3)	12 (7.1)
Education, n (%)			
Less than high school	105 (28.2)	79 (26.2)	53 (31.4)
High school graduate, no college	62 (16.6)	49 (16.3)	29 (17.2)
Some college or associate's degree	38 (10.2)	25 (8.3)	9 (5.3)
Bachelor's degree or higher	78 (20.9)	59 (19.6)	35 (20.7)
Unknown/not available	90 (24.1)	89 (29.6)	43 (25.4)
Insurance type, n (%)			
N	373	301	169
None	89 (23.9)	93 (30.9)	60 (35.5)
Private	20 (5.4)	8 (2.7)	13 (7.7)
Public/government	256 (68.6)	192 (63.8)	96 (56.8)
Mixed	6 (1.6)	7 (2.3)	0 (0.0)
Other	2 (0.5)	1 (0.3)	0 (0.0)

Abbreviations: ET, essential thrombocythemia; MF, myelofibrosis; PV, polycythemia vera.

^a^ In the clinical study report, % patients with Intermediate‐1 and Intermediate‐2 risks were not interpreted appropriately, and hence not captured in the table.

^b^ The percentages do not represent the true incidence as the sites were not asked if they routinely perform these mutational analyses.

^c^ CALR and JAK2 exon 12 mutations were not analyzed in majority of patients/centers.

**Table 2:** Patient disposition

Disposition, n (%)	Overall (N = 1044)
Patients enrolled in the study, n	1044
Patients included (full analysis set)	884 (84.7)
Patients excluded due to protocol deviation	160 (15.3)
Patients completing the study	462 (52.3)
Patients discontinuing the study before completion	422 (47.7)
Reason for discontinuation	
Adverse events	0
Lost to follow‐up	176 (41.7)
Withdrew consent	101 (23.9)
Disease progression	2 (0.5)
Death	57 (13.5)
Other	86 (20.4)
Reasons for exclusion due to PD	
Inclusion/exclusion criteria not met	1 (0.6)
Other	159 (99.4)
Patient did not sign an updated ICF version during the study	158 (98.8)
Original paper medical records of the patient were missing at the site	1 (0.6)

Abbreviations: ICF, informed consent form; PD, disease progression.

### 3.2 MPN type and clinical characteristics at initial diagnosis

MPN subtypes were remarkably different in the Philippines and India: the proportion of patients diagnosed with MF was higher (32.6% and 40.2%, respectively) and those diagnosed with ET was less prevalent (20.9% and 18.6%, respectively) compared with the total distribution in this study. In China, patients with ET were more frequent (58.4%) and patients with PV were less common (19.5%). In Taiwan, patients with ET were more frequent (49.1%) and patients with MF were less frequent (11.8%). In Qatar, the proportion of patients with unclassified MPNs was substantially higher (36.0%) than in other participating countries, and only a few patients (4.0%) were enrolled with an initial diagnosis of MF. Overall, 69.4% of ET patients (N = 373), 53.5% of PV patients (N = 301), and 37.9% (N = 169) of MF patients were diagnosed incidentally before the onset of signs/symptoms, while 29.8% of ET patients, 43.5% of PV patients, and 59.8% of MF patients presented with signs and/or symptoms at diagnosis.

### 3.3 Prevalence and incidence of MPNs by country

The estimates of annual hospital‐based occurrence of existing and newly diagnosed cases of MPNs per 100 000 patients visiting MERGE sites differed substantially between countries, and varied considerably over time in some countries (Appendix 2). Among 100 000 patients who visited the participating hospitals from 2013 to 2017, between 57 and 81 patients with Ph‐MPNs were seen annually by the participating physicians. The highest rate of Ph‐MPNs was reported at the study start, followed by a reduction in the number of patients with Ph‐MPNs seen/reported in the subsequent years until 2016 to 64 patients with Ph‐MPNs per 100 000 hospital patients in the same year. Annually, between 12 and 15 newly diagnosed patients with Ph‐MPNs were seen by physicians per 100 000 hospital patients during the study period, starting at 14 incident cases in 2013 that reduced to 12 cases per 100 000 hospital patients in 2016, showing a similar decreasing trend.

### 3.4 Treatment

During the study, 64% of patients received hydroxyurea, 15% received interferon, 15% received JAK inhibitors, and 10% received anagrelide either alone or in combination. Overall (N = 635), the median duration of treatment was estimated as 5.7 months (Q1: 1.4 months; Q3: 19.5 months). The most common medications used for first‐line therapy across all indications were aspirin (32.6%) and hydroxyurea (53.8%), administered as monotherapy or in combination. Hydroxyurea was the most frequently used first‐line treatment prescribed in 39.1% of patients with MF vs 53.8% and 61.2% of patients with ET and PV, respectively. Aspirin was reported as the second most frequently used first‐line therapy in 17.2% of patients with MF vs 36.8% and 37.7% of patients with ET and PV, respectively. Other common first‐line therapies were anagrelide (10%) and interferon (9%) in patients with ET, cytoreductive therapy (6%) and clopidogrel (6%) in patients with PV, and JAK2 inhibitors (15%) in patients with MF. More than 75% of the induction therapies were monotherapies, with less than 3% of patients receiving three or more drug combinations as primary treatment. Patients with MF often received monotherapy (81%) than the other patients with MPNs. The median duration of first‐line therapy was approximately 6 months (95% CI, 1‐21 months), and first‐line therapy discontinuation rates were 35%, 36%, and 30% in ET, PV, and MF patients, respectively. Interferon was used in 8% of patients in the second‐line setting. JAK2 inhibitors were more frequently (14%‐17%) used in the second‐ and third‐line settings. A considerably higher proportion of patients with MF received JAK2 inhibitors (14.6%), androgens (9.9%), and immunomodulatory agents (8.6%) as the first LOT compared with patients diagnosed with ET and PV. The longest median (Q1‐Q3) time from diagnosis until treatment initiation was observed in patients with MF (N = 149): 1.6 (0.1‐10.6) months vs 0.0 (0.0‐5.1) months in patients with ET (N = 353) and 0.3 (0.0‐10.1) months in patients with PV (N = 272) (Kruskal‐Wallis *P*‐value < .001). During the study period, the most common non‐pharmacological intervention was red cell transfusion in patients with MF and ET, and phlebotomy in patients with PV. Splenectomy (n = 2) and stem cell transplantation (n = 4) were rarely employed (six MF patients).

A total of 75.6% of patients received medications not related to MPNs. Nearly 20% of patients with MPNs received at least one of the following concomitant medications: paracetamol, antiplatelet agents (excluding heparin), anti‐gout preparations, dihydropyridine derivatives, and proton pump inhibitors. Although patients with ET reported a higher prevalence of comorbidities than patients with MF, a lower proportion of them received concomitant medications (71.0%) compared with patients with MF (75.7%). The treatment pattern of patients with MF differed from the rest of the patients with MPNs: proton pump inhibitors and folic acid and derivatives were more commonly used, while dihydropyridine derivatives and platelet aggregation inhibitors were less frequently used.

### 3.5 Splenomegaly

Overall, 30.0% of the patients were assessed for splenomegaly at baseline. Among them, 43.9% of patients recorded an increase in spleen size at 6 months after baseline (N = 82). The proportion of patients experiencing an increase in spleen size since last visit increased during follow‐up, and was estimated to be 65.6% at 24 months after baseline (N = 32). Post‐baseline, patients continued to develop palpable splenomegaly (12.7% at 6 months, 10.7% at 12 months, 7.2% at 18 months, and 6.8% at 24 months). The mean (standard deviation [SD]) spleen size as measured by palpation varied from 8.2 (5.53) cm at baseline (N = 226) to 11.3 (6.19) cm at 18 months after baseline (N = 27), decreasing to 8.3 (4.47) cm at 24 months after baseline (N = 24).

### 3.6 Bone marrow biopsy and peripheral blood smear

In the 12 months prior to baseline, bone marrow biopsy/aspirate or peripheral blood smear was performed in 333 patients, representing 37.7% of the full analysis set (N = 884). The most frequently recorded fibrosis grade was grade 2, observed in 8.7% of patients. A similar proportion of patients was observed across reticulin and collagen grades 0 to 3 (between 8.4% and 9.6%), while only 3.6% of patients presented with grade 4. Mean (SD) blast count was estimated to be 1.0% (1.72%) within 12 months prior to baseline (Appendix 3).

### 3.7 MPN‐SAF TSS score by initial diagnosis and by study visit

**Figure 1:**
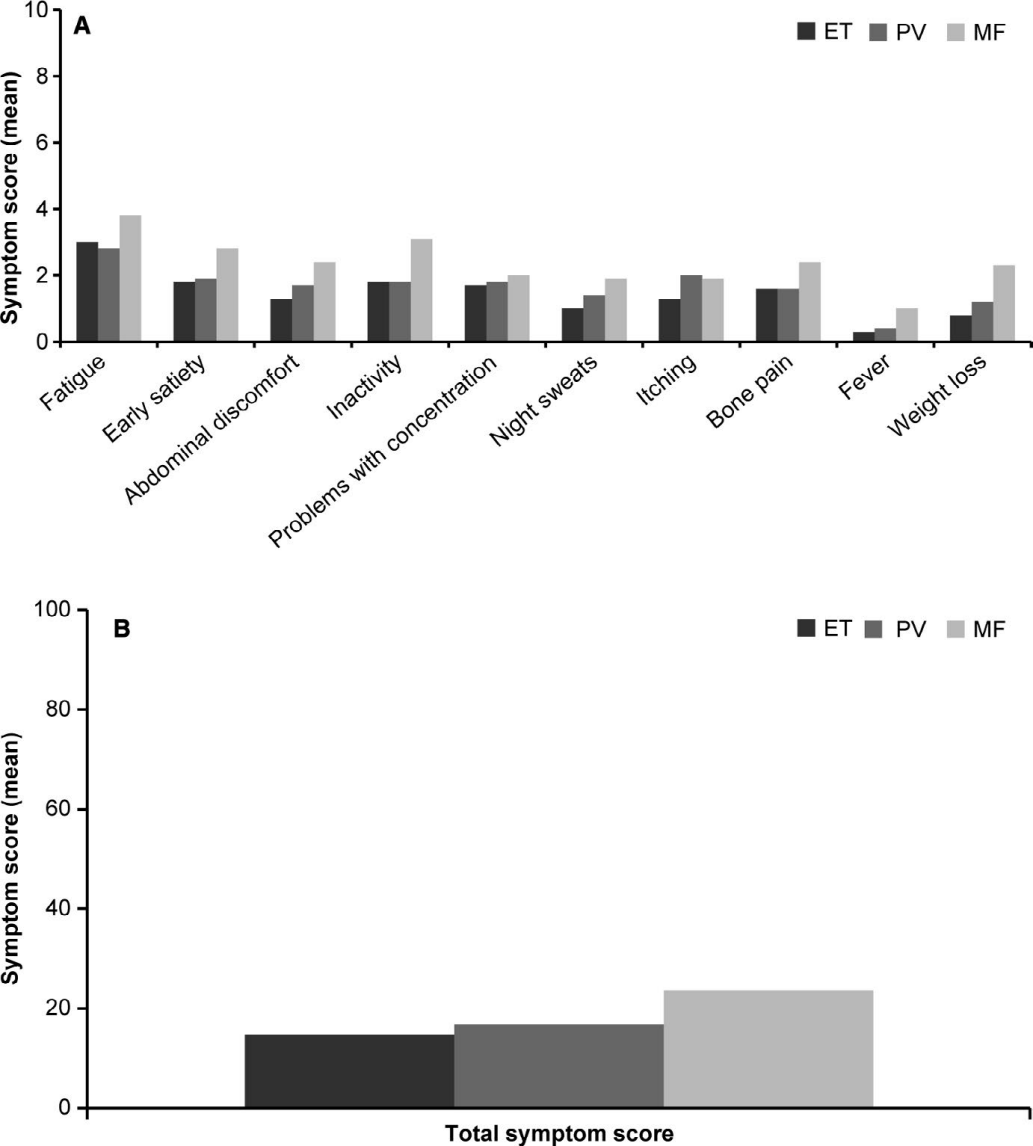
MPN‐associated symptom assessment by initial diagnosis^a^. (A) Individual symptom score and (B) Total symptom score. ET, essential thrombocythemia; MF, myelofibrosis; MPN, myeloproliferative neoplasm; PV, polycythemia vera. ^a^A MPN Symptom Assessment Form Total Symptom Score (MPN‐SAF TSS) ≥ 20, a worst individual item score > 5, or combined criteria of both warrant treatment

**Figure 2:**
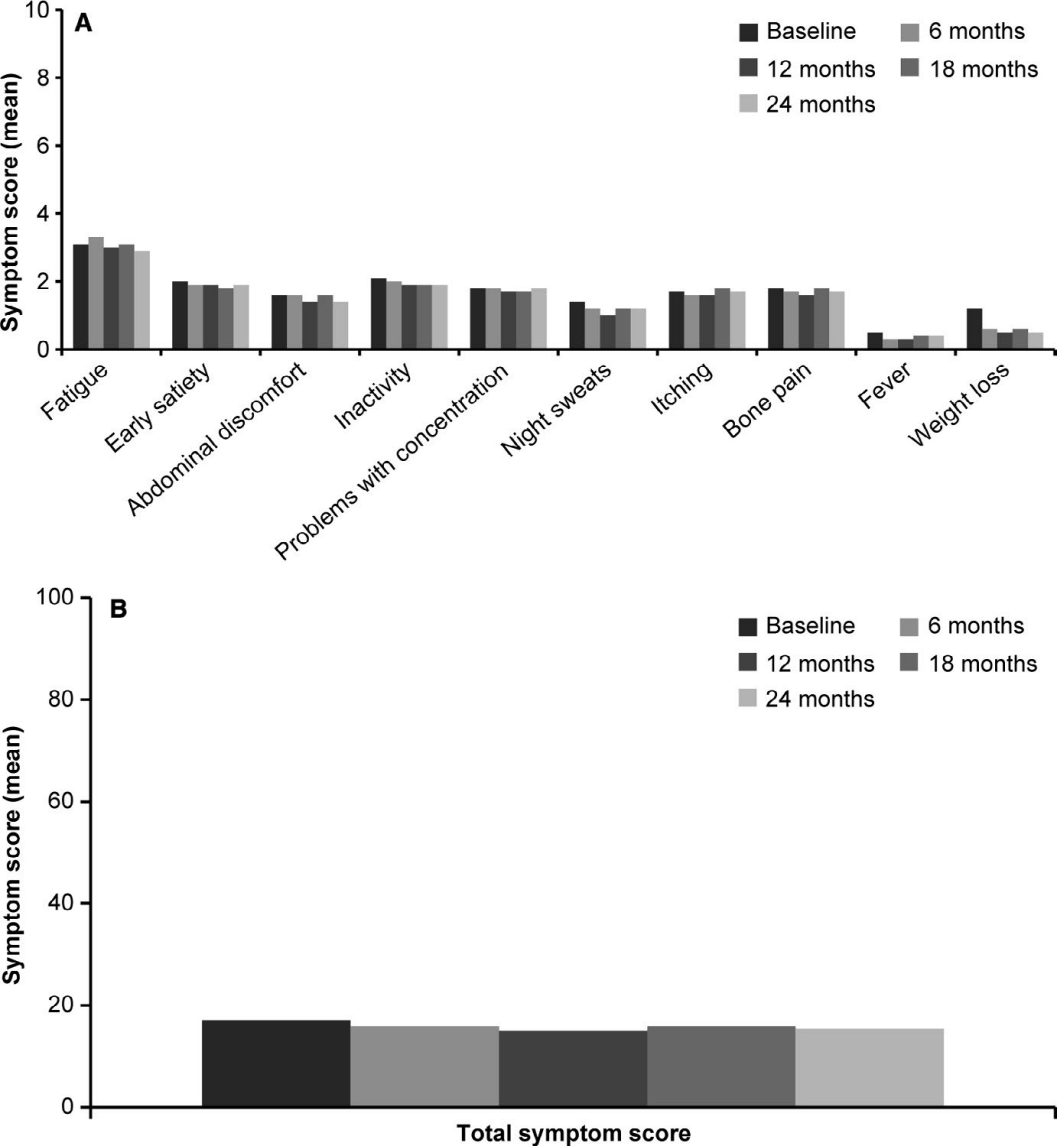
MPN‐associated symptom assessment by study visit^a^. (A) Individual symptom score and (B) Total symptom score. MPN, myeloproliferative neoplasm. ^a^A MPN Symptom Assessment Form Total Symptom Score (MPN‐SAF TSS) ≥20, a worst individual item score > 5, or combined criteria of both warrant treatment

At baseline, the proportion of patients presenting with at least one of the symptoms was higher in the MF group (96.4%) compared with the total patients with MPNs (91.8%). The most commonly reported symptom in patients with MPNs was fatigue (71.4%). Other reported symptoms included inactivity (49.9%), problems with concentration (48.2%), early satiety (48.1%), bone pain (43.1%), abdominal discomfort (42.7%), pruritus (41.0%), night sweats (34.4%), unintentional weight loss (31.7%), and fever (14.1%). The maximum individual symptom score was 10, while the TSS was 100. The TSS (mean [SD]) at baseline was highest in patients with MF (23.5 [17.47]) compared with patients with ET (14.6 [14.26]) and PV (16.6 [14.84]) (Figure 1). Consistent symptom patterns have been observed among patients with ET, PV, and MF, with fatigue being the most commonly reported symptom and fever being the least reported one. Early satiety, abdominal discomfort, inactivity, night sweats, bone pain, fever, and unintentional weight loss were significantly more common and intense in patients with MF (*P* < .001). During study duration and follow‐up, the TSS remained more or less constant at subsequent visits. The results did not show a clear temporal trend either in the total score or in the individual symptoms during the follow‐up (Figure 2).

### 3.8 Patient and physician assessment of MPN‐associated symptoms

Although physicians reported a lower proportion of symptoms than that self‐reported by patients, there was a better agreement between physicians and patients for pruritus and night sweats (Kappa, 0.61‐0.80) (Table 3). A moderate level of agreement (Kappa, 0.41‐0.60) was observed for all other symptoms: bone pain, abdominal discomfort, fever, unintentional weight loss, early satiety, fatigue, inactivity, and problems with concentration. Across MPN subtypes at initial diagnosis, inter‐rater agreement was lowest for patients with MF vs patients with ET or PV.

**Table 3:** Patient and physician perspectives on MPN‐associated symptoms at baseline

	Overall		Cohen's Kappa coefficient^b^	ET at initial diagnosis^a^		PV at initial diagnosis^a^		MF at initial diagnosis^a^	
	Patient (N = 884) n (%)	Physician (N = 884) n (%)		Patient (N = 373) n (%)	Physician (N = 373) n (%)	Patient (N = 301) n (%)	Physician (N = 301) n (%)	Patient (N = 169) n (%)	Physician (N = 169) n (%)
Fatigue (Weariness, Tiredness)									
N	870	884		368	373	297	301	166	169
Patients with score > 0^c^	621 (71.4)	424 (48.0)	0.483	262 (71.2)	182 (48.8)	202 (68.0)	141 (46.8)	129 (77.7)	88 (52.1)
Missing	14	0		5	0	4	0	3	0
Filling up quickly when you eat (early satiety)									
N	874	884		369	373	297	301	167	169
Patients with score > 0^c^	420 (48.1)	225 (25.5)	0.527	163 (44.2)	93 (24.9)	131 (44.1)	70 (23.3)	102 (61.1)	56 (33.1)
Missing	10	0		4	0	4	0	2	0
Abdominal discomfort									
N	874	884		367	373	298	301	168	169
Patients with score > 0^c^	373 (42.7)	230 (26.0)	0.577	138 (37.6)	81 (21.7)	120 (40.3)	74 (24.6)	96 (57.1)	70 (41.4)
Missing	10	0		6	0	3	0	1	0
Inactivity									
N	875	884		369	373	298	301	167	169
Patients with score > 0^c^	437 (49.9)	228 (25.8)	0.481	171 (46.3)	101 (27.1)	138 (46.3)	69 (22.9)	107 (64.1)	54 (32.0)
Missing	9	0		4	0	3	0	2	0
Problems with concentration									
N	876	884		369	373	298	301	168	169
Patients with score > 0^c^	422 (48.2)	206 (23.3)	0.469	165 (44.7)	90 (24.1)	144 (48.3)	71 (23.6)	91 (54.2)	41 (24.3)
Missing	8	0		4	0	3	0	1	0
Night sweats									
N	876	884		369	373	298	301	168	169
Patients with score > 0^c^	301 (34.4)	191 (21.6)	0.648	100 (27.1)	65 (17.4)	106 (35.6)	68 (22.6)	77 (45.8)	56 (33.1)
Missing	8	0		4	0	3	0	1	0
Itching (pruritus)									
N	874	884		368	373	298	301	168	169
Patients with score > 0^c^	358 (41.0)	239 (27.0)	0.649	124 (33.7)	95 (25.5)	148 (49.7)	102 (33.9)	70 (41.7)	37 (21.9)
Missing	10	0		5	0	3	0	1	0
Bone pain (diffuse, not joint pain or arthritis)									
N	872	884		366	373	298	301	168	169
Patients with score > 0^c^	376 (43.1)	221 (25.0)	0.582	147 (40.2)	99 (26.5)	115 (38.6)	72 (23.9)	94 (56.0)	46 (27.2)
Missing	12	0		7	0	3	0	1	0
Fever (>37.8°C)									
N	874	884		368	373	298	301	168	169
Patients with score > 0^c^	123 (14.1)	70 (7.9)	0.560	30 (8.2)	20 (5.4)	41 (13.8)	25 (8.3)	48 (28.6)	25 (14.8)
Missing	10	0		5	0	3	0	1	0
Unintentional weight loss in the last 6 months									
N	872	884		367	373	297	301	168	169
Patients with score > 0^c^	276 (31.7)	164 (18.6)	0.556	82 (22.3)	46 (12.3)	94 (31.6)	62 (20.6)	88 (52.4)	53 (31.4)
Missing	12	0		6	0	4	0	1	0

Abbreviations: ET, essential thrombocythemia; MF, myelofibrosis; MPN, myeloproliferative neoplasm; PV, polycythemia vera.

^a^ Initial diagnosis: diagnosis reported retrospectively at baseline visit; corresponded to the first MPN subtype diagnosed (at diagnosis date). Overall column also includes patients with unclassified MPNs.

^b^ Only Cohen's Kappa coefficient for overall sample was calculated.

^c^ For physician's column, if a particular MPN disease‐associated symptom was ticked in the case record form, it was counted in “patients with score > 0” row.

### 3.9 MPN‐SAF TSS total score and selected covariates

In a multivariate adjusted mixed effect regression model, female gender, type of MPN (MF), presence of comorbidities, and lower baseline hemoglobin were independently and positively associated with the symptom score values (Table 4).

**Table 4:** Mixed effect regression model (MPN‐SAF TSS Total Score)

Covariate	Category	Unadjusted model^a^			Adjusted model^b^	
		Group Mean	ß (95% CI)	*P*‐value	ß (95% CI)	*P*‐value
Fixed parts						
Age	<65 y	16.38	Reference		Reference	
	≥65 y	15.99	−0.39 (2.34, 1.56)	.695	—	—
Sex	Male	15.16	Reference		Reference	
	Female	17.38	2.22 (0.43, 4.01)	.015	2.53 (0.58, 4.48)	.011
Weight at baseline		17.34	−0.02 (−0.10, 0.06)	.595	—	—
Height at baseline		19.17	−0.02 (−0.14, 0.10)	.742	—	—
Family history of MPNs	No	16.90	Reference		Reference	
	Yes	17.71	0.81 (−10.91, 12.54)	.891		
Comorbidities	No	15.60	Reference		Reference	.043
	Yes	17.38	1.78 (−0.07, 3.64)	.059	2.07 (0.06, 4.08)	
Type of initial MPN	ET	14.56	Reference		Reference	
	PV	15.24	0.68 (−1.34, 2.69)	.511	0.03 (−2.25, 2.32)	.976
	MF	22.54	7.97 (5.54, 10.41)	<.001	4.89 (1.87, 7.90)	.002
	Unclassified	13.97	−0.59 (−4.93, 3.74)	.788	−0.84 (−5.50, 3.82)	.723
Hemoglobin baseline level		17.89	−0.02 (−0.03, −0.01)	<.001	−0.03 (−0.05, −0.02)	<.001
Platelet count baseline level		18.21	−0.00 (−0.01, −0.00)	.003	−0.00 (−0.01, 0.00)	.135
ECOG PS at baseline		16.67	1.49 (0.23, 2.75)	.021	1.24 (−0.12, 2.61)	.074
Time since diagnosis until MPN treatment initiation (months)		15.80	0.03 (0.01, 0.06)	.019	0.01 (−0.02, 0.04)	.392

Abbreviations: CI, confidence interval; ECOG, Eastern Cooperative Oncology Group; ET, essential thrombocythemia; MF, myelofibrosis; MPN, myeloproliferative neoplasm; MPN‐SAF TSS, MPN Symptom Assessment Form Total Symptom Score; PS, performance status; PV, polycythemia vera.

^a^ Unadjusted models using a univariate mixed model.

^b^ Adjusted model using a multivariate mixed model; only coefficients for variables included in the final model were described (*P* < .2).

### 3.10 Health‐care resource utilization

Overall, 848 patients with MPNs had utilized health‐care resources at different time points: 12 months prior to baseline and first and second years of follow‐up. At least one inpatient visit was reported in 24.3% of the patients, with a mean duration of 11.1 days (Table 5). Outpatient visits (95.5%) were the most commonly used health‐care resource, without any remarkable change during the follow‐up period. Patients with ET had a lower number of inpatient visits (mean [SD]: 0.9 [0.77] days) and patients with MF had more outpatient visits (mean [SD]: 5.2 [3.17] days) on an average, compared with the entire MPN group.

**Table 5:** Health‐care resource utilization

	Overall^a^ (N = 884)	ET at initial diagnosis^a^ (N = 373)	PV at initial diagnosis^a^ (N = 301)	MF at initial diagnosis^a^ (N = 169)	*P*‐value
Has the patient used health‐care resources in connection with their Ph‐MPN disease? n (%)					
N	884	373	301	169	.451
No	36 (4.1)	12 (3.2)	14 (4.7)	9 (5.3)	
Yes	848 (95.9)	361 (96.8)	287 (95.3)	160 (94.7)	
Number of inpatient visits					
N	214	84	58	71	.003
Mean (SD)	1.3 (1.76)	0.9 (0.77)	1.5 (2.17)	1.7 (2.13)	
Number of total days per inpatient visit					
N	155	50	48	56	.081
Mean (SD)	11.1 (12.16)	9.1 (10.01)	11.3 (9.42)	12.9 (15.50)	
Number of outpatient visits					
N	844	360	285	159	<.001
Mean (SD)	4.4 (3.20)	4.2 (3.00)	4.3 (3.41)	5.2 (3.17)	
Number of hospice care visits					
N	17	10	4	3	.666
Mean (SD)	0.9 (0.93)	0.7 (0.82)	1.3 (1.26)	1.0 (1.00)	
Number of total days per hospice care visit					
N	6	3	1	2	.304
Mean (SD)	6.9 (5.73)	9.3 (7.02)	0.0 (‐)	6.8 (0.35)	
Number of day care visit					
N	58	19	26	12	.016
Mean (SD)	1.7 (2.18)	0.8 (1.68)	1.9 (1.92)	2.9 (2.96)	
Number of total days per day care visit					
N	32	5	19	7	.241
Mean (SD)	3.1 (2.39)	3.3 (1.88)	2.7 (2.26)	4.3 (2.99)	
Number of emergency room visits					
N	119	55	30	30	.609
Mean (SD)	1.1 (1.75)	0.9 (1.45)	0.9 (1.01)	1.7 (2.63)	

Abbreviations: ET, essential thrombocythemia; MF, myelofibrosis; MPN, myeloproliferative neoplasm; Ph−, Philadelphia chromosome‐negative; PV, polycythemia vera; SD, standard deviation.

^a^ Initial diagnosis: diagnosis reported retrospectively at baseline visit; corresponds to the first Ph‐MPN sub‐type diagnosed (at diagnosis date). Overall coulmn also includes patients with unclassified MPN.

### 3.11 Time to disease progression

The time to disease progression since initial diagnosis of MPNs was only available for five patients, including one patient initially diagnosed with ET and two patients with MF. Overall, the mean (SD) time since diagnosis of MPNs until disease progression was 14.1 (11.75) months. The initial diagnosis of MPNs was found to be significantly associated with disease progression (*P* = .011). Patients with unclassified MPNs had a higher risk of disease progression compared with patients with ET (hazard ratio [HR] 42.68; 95% CI, 3.77‐482.9).

## 4 DISCUSSION

MERGE is the first noninterventional registry study that provides epidemiological data on the distribution of MPNs and their diagnosis, management, and treatment patterns in 1044 patients enrolled from 54 sites in 17 countries in Asia, including the Middle East, Turkey, and Algeria. Patients included in the analysis were followed for up to 2 years, providing real‐world data on the natural course of MPNs, patterns of diagnosis, treatment of MPNs, and long‐term clinical outcomes. The prevalence of MPNs has been reported to be between 14.2 and 20.8 patients per 100 000 inhabitants.^12^, ^13^ The incidence of MPNs is largely unknown in most areas; however, studies performed in the United States and Europe have shown incidence rates of 3.1 and 2.7 cases per 100 000 persons per year, respectively.^14^, ^15^ Due to the methodological characteristics of the study, and the limitations regarding assessing the incidence and prevalence of Ph‐MPNs, the frequency estimates are not directly comparable with the incidence and prevalence estimations reported in the literature.

Overall, just under half of the patients discontinued before study completion, with the most common reason being the patient lost to follow‐up. In the controlled clinical studies for MF (COMFORT‐I,^16^ COMFORT‐II,^17^) and PV (RESPONSE,^18^), a lower proportion of patients lost to follow‐up and the rate of discontinuation decreased over time. This may be attributed to a more intense monitoring and follow‐up in the controlled clinical studies.

The results from our study showed slight differences in the distribution pattern of MPN subtypes. While the most frequent MPN subtype was PV (between 44.2% and 52.9%) in the United States, Europe, and a few Asian countries,^12^, ^19^, ^20^ 34% of patients had PV in our study population. The country‐specific differences in MPN subtypes at initial diagnosis can further explain the observed differences; however, limited conclusions can be drawn for some countries due to the small sample of patients contributing to the overall analysis. The high incidence of patients with unclassified MPNs in Qatar is likely due to a large number of former patients who may not be evaluated completely for various reasons. As most centers are tertiary, a higher incidence of MF is noticed in India where symptomatic patients are more likely to access tertiary care facilities.

The baseline characteristics of the patients included in this study were mainly as expected for patients with MPNs and are similar to a previously published study.^1^ The median age reported in this study appears to be lower than that reported in Norway,^1^ but almost similar to that of other developed countries reported from Landmark survey.^1^

Patients with PV were at a higher risk of cardiovascular comorbidity. The risk factors for cardiovascular and thrombotic events in patients with PV included advanced age, a history of thrombosis, elevated hematocrit, and leukocytosis.^21^ One‐third of the patients in this study had at least one comorbidity. Patients with PV were more likely to have any concomitant disease compared with other patients. Patients with MF with the worst prognosis had fewer comorbidities, with the most pronounced difference in cardiovascular comorbidities.

The most frequent first‐line treatment in all Ph‐MPN subtypes was hydroxyurea. After the initial diagnosis of Ph‐MPNs, more than half of the patients received hydroxyurea as the first LOT, which is more commonly used among patients with PV at initial diagnosis and less frequently among patients with MF. The use of JAK2 inhibitors increased considerably from the first LOT to second‐line and third‐line and beyond. This might indicate a slow and gradually increasing availability of newer agents in these countries, despite clear evidence of the superiority of newer agents over conventional therapies from randomized controlled trials.^22^

Overall, MPNs were diagnosed on an average 3.5 years before the study entry (baseline). Less than half of the patients had signs and symptoms at diagnosis. This could be attributed to the insidious nature and lack of specific symptoms of the disease; however, lack of disease awareness in these countries cannot be ruled out. The clinical manifestations included polycythemia, anemia, leukocytosis, thrombocytosis, fatigue, and hepatosplenomegaly, and these varied by the MPN subtype. This is not surprising given the heterogeneous nature of MPNs.^23^

Patients with MF more frequently experienced MPN disease‐associated symptoms compared with all patients, with fatigue being the most commonly reported symptom, which is in line with the global MPN Landmark survey.^1^ Similar to global MPN Landmark survey,^1^ discordance between physician and patient perception of symptom assessment was observed in this study, indicating that in addition to spleen and hematological investigations, a more systematic assessment, such as the use of MPN‐SAF TSS, could help to better evaluate patients’ symptoms and understand the disease and its treatment burden.

Our study highlights a growing need to educate patients for more symptom awareness and to educate physicians regarding the utilization of symptom assessment tools for initial assessment of patients for accurate evaluation of symptom burden.

### 4.1 Limitations

The estimations of incidence and prevalence were based on retrospective data collection. A limitation is that data integrity and quality issues often arise in the collection of retrospective event data; selection bias is presumably present, and moreover, potential for corrective actions is limited. The data on the annual number of patients visiting a particular hospital were collected and used in the calculations as a proxy of the population at risk, instead of the catchment population of each of the hospitals. In some countries, local regulations did not allow sites to collect data about the patients who did not sign the consent form, and therefore, those countries were excluded from the prevalence/incidence calculations. The estimates of annual hospital‐based occurrence of MPNs per 100 000 patients differed substantially between countries, and varied considerably over time in some countries. This can be due to the retrospective nature of data collection, subject to under‐ or overestimation of MPN cases and total patients visiting MERGE hospitals. The use of hospital‐based prevalence and incidence rates can highly underestimate the size of the population at risk, with consequent overestimation of the hospital‐based estimates compared with population‐based estimates.

## 5 CONCLUSION

MERGE registry provided a description of the current clinical practice management landscape of MPNs for countries in Asia for mutation testing, investigations, treatments, and compliance/adherence. The data from the MERGE registry showed that patients with MPNs have a severe disease burden and reduced QOL. Despite being on treatment, patients with MPNs had a substantial symptom burden, which did not change over the course of the study. A discordance between physician and patient perception of symptom assessment was observed in this study, indicating that in addition to spleen and hematological investigations, a more systematic assessment such as the use of validated tools such as MPN‐SAF TSS could help to better evaluate patients’ symptoms and understand the disease and its treatment burden. Being female, having a diagnosis of MF, the presence of comorbidities, and lower baseline hemoglobin were all associated with a significantly higher TSS. Hydroxyurea and aspirin, as monotherapy or in combination, were the most commonly prescribed first LOT. JAK2 inhibitors were used among the second‐line/third‐line options mainly for MF. To further understand the impact of differences in geographical clinical practice and impact on patient management and outcomes, the outcomes of MERGE registry may need to be compared with that of disease burden and demographics of MPNs from other regions/countries.

## CONFLICT OF INTEREST

MAY: Novartis: Research funding; AT: Novartis: Research funding, honoraria, and consultation fee. TS: Novartis and Roche: Consultancy. SJK: Novartis: Honoraria. GR: Employee of IQVIA ‐ doing consultancy for Novartis. IS: Novartis: Employment and equity ownership. AS: Novartis Pharma AG: Employment and equity ownership. RSW: Novartis: Consultancy, membership on an entity's Board of Directors or advisory committees, research funding and speakers bureau. VM, HAH, TFT, ZX, WD, and JL have no conflict of interest to disclose.

## AUTHOR CONTRIBUTIONS

MAY, AT, IS, RSW, and SJK contributed to the concept and design. Contributions to data collection were done by MAY, AT, VM, HAH, TS, TFT, ZX, SJK, WD, JL, and RSW. Data analysis and interpretation were contributed by MAY, AT, VM, HAH, TFT, ZX, SJK, GR, IS, AS, and RSW. All authors drafted, reviewed, and approved the manuscript and accept accountability for the same.

## Data Availability

The datasets generated during and/or analyzed during the current study are available from the corresponding author on reasonable request.

## Appendix 1 Patient distribution per site and country

**Table:**

Country/location	Recruitment sites	Patients enrolled	Patients included in the analysis, n (%)
Total	54	1044	884
China	6	219	185 (20.9)
Taiwan	4	115	110 (12.4)
India	10	127	102 (11.5)
Turkey	5	104	90 (10.2)
South Korea	7	80	78 (8.8)
Pakistan	2	75	70 (7.9)
Qatar	1	61	50 (5.7)
Philippines	2	63	43 (4.9)
Israel	3	60	32 (3.6)
Malaysia	1	25	24 (2.7)
Algeria	5	24	23 (2.6)
Vietnam	1	24	22 (2.5)
Lebanon	1	22	16 (1.8)
Hong Kong	2	15	15 (1.7)
UAE	2	18	14 (1.6)
Singapore	1	8	7 (0.8)
Kuwait	1	4	3 (0.3)

## Appendix 2 Prevalent and incident cases of MPNs by year

**Table:**

	Total number of patients used for the estimation of hospital‐based prevalent cases per 100 000	Total number of cases	Prevalent cases per 100 000 (95% CI)	Total number of patients used for the estimation of hospital‐based incident cases per 100 000	Total number of new cases	Incident cases per 100 000 per year (95% CI)
2013	16 852 913	13 570	80.52 (79.17, 81.89)	18 392 128	2590	14.08 (13.54, 14.64)
2014	17 773 876	12 485	70.24 (69.02, 71.49)	19 349 107	2857	14.77 (14.23, 15.32)
2015	23 326 640	13 219	56.67 (55.71, 57.64)	23 326 640	2764	11.85 (11.41, 12.30)
2016	20 161 286	12 932	64.14 (63.04, 65.26)	22 880 844	2757	12.05 (11.60, 12.51)
2017^a^	800 000	531	66.38 (60.85, 72.27)	800 000	141	17.63 (14.84, 20.79)

^a^ Data for the year 2017 were provided by only one site, located in Turkey.

## Appendix 3 Bone marrow biopsy/aspirate or peripheral blood smear by study visit

**Table:**

	Within 12 mo prior to baseline (N = 884)	At 6 mo after baseline (N = 884)	At 12 mo after baseline (N = 884)	At 18 mo after baseline (N = 884)	At 24 mo after baseline (N = 884)
Bone marrow biopsy/aspirate or peripheral blood smear performed, n (%)					
N	884	599	506	456	424
Yes	333 (37.7)	43 (7.2)	27 (5.3)	20 (4.4)	25 (5.9)
No	551 (62.3)	556 (92.8)	479 (94.7)	436 (95.6)	399 (94.1)
Fibrosis grade, n (%)					
Not assessed	197 (59.2)	40 (93.0)	24 (88.9)	18 (90.0)	20 (80.0)
Grade not available	47 (14.1)	0 (0.0)	2 (7.4)	0 (0.0)	2 (8.0)
Grade 0	18 (5.4)	0 (0.0)	0 (0.0)	0 (0.0)	1 (4.0)
Grade 1	21 (6.3)	1 (2.3)	0 (0.0)	0 (0.0)	2 (8.0)
Grade 2	29 (8.7)	1 (2.3)	1 (3.7)	0 (0.0)	0 (0.0)
Grade 3	21 (6.3)	1 (2.3)	0 (0.0)	2 (10.0)	0 (0.0)
Reticulin and collagen grade, n (%)					
Not assessed	186 (55.9)	39 (90.7)	23 (85.2)	16 (80.0)	19 (76.0)
Grade not available	17 (5.1)	0 (0.0)	2 (7.4)	0 (0.0)	0 (0.0)
Grade 0	29 (8.7)	1 (2.3)	1 (3.7)	0 (0.0)	2 (8.0)
Grade 1	32 (9.6)	0 (0.0)	0 (0.0)	0 (0.0)	1 (4.0)
Grade 2	29 (8.7)	2 (4.7)	1 (3.7)	0 (0.0)	3 (12.0)
Grade 3	28 (8.4)	1 (2.3)	0 (0.0)	4 (20.0)	0 (0.0)
Grade 4	12 (3.6)	0 (0.0)	0 (0.0)	0 (0.0)	0 (0.0)
